# T Cell Receptor Signal Initiation Induced by Low-Grade Stimulation Requires the Cooperation of LAT in Human T Cells

**DOI:** 10.1371/journal.pone.0015114

**Published:** 2010-11-30

**Authors:** Shen Dong, Béatrice Corre, Konstantina Nika, Sandra Pellegrini, Frédérique Michel

**Affiliations:** 1 Unit of Cytokine Signaling, Department of Immunology, Institut Pasteur, Paris, France; 2 CNRS URA 1961, Paris, France; 3 Sir William Dunn School of Pathology, University of Oxford, Oxford, United Kingdom; New York University, United States of America

## Abstract

**Background:**

One of the earliest activation events following stimulation of the T cell receptor (TCR) is the phosphorylation of the immunoreceptor tyrosine-based activation motifs (ITAMs) within the CD3-associated complex by the Src family kinase Lck. There is accumulating evidence that a large pool of Lck is constitutively active in T cells but how the TCR is connected to Lck and to the downstream signaling cascade remains elusive.

**Methodology/Principal Findings:**

We have analyzed the phosphorylation state of Lck and Fyn and TCR signaling in human naïve CD4^+^ T cells and in the transformed T cell line, Hut-78. The latter has been shown to be similar to primary T cells in TCR-inducible phosphorylations and can be highly knocked down by RNA interference. In both T cell types, basal phosphorylation of Lck and Fyn on their activatory tyrosine was observed, although this was much less pronounced in Hut-78 cells. TCR stimulation led to the co-precipitation of Lck with the transmembrane adaptor protein LAT (linker for activation of T cells), Erk-mediated phosphorylation of Lck and no detectable dephosphorylation of Lck inhibitory tyrosine. Strikingly, upon LAT knockdown in Hut-78 cells, we found that LAT promoted TCR-induced phosphorylation of Lck and Fyn activatory tyrosines, TCRζ chain phosphorylation and Zap-70 activation. Notably, LAT regulated these events at low strength of TCR engagement.

**Conclusions/Significance:**

Our results indicate for the first time that LAT promotes TCR signal initiation and suggest that this adaptor may contribute to maintain active Lck in proximity of their substrates.

## Introduction

The Src family protein tyrosine kinases (PTK), Lck and Fyn, play a key role in the adaptive immune system by controlling T cell development, activation and functions. Upon TCR triggering by cognate peptide-MHC, Lck primarily controls T cell activation by phosphorylating CD3 ITAM motifs and by amplifying the signal through the co-receptor CD4 to which it is associated [1], [2], [3]. In addition, Lck enhances TCR-induced Zap-70 activation by phosphorylating two critical residues, the positive regulatory Tyr319 situated on the interdomain B and the activatory Tyr493 within the activation loop of the kinase domain [4], [5]. Zap-70 in turn phosphorylates the transmembrane scaffold protein LAT. By controlling the cooperative assembly of signaling complexes that play positive or negative roles, LAT ensures signal diversification [6], [7], [8], [9]. The Lck homologue, Fyn, is well expressed in mature T cells and participates to CD3 phosphorylation [10]. However, its role in TCR signaling is less evident than that of Lck. Indeed, Fyn is partially redundant with Lck and is involved in a feedback loop that dampens Lck activation [11], [12].

Despite the description of several regulators of Lck and Fyn, how the TCR communicates with these kinases to initiate the signaling cascade remains unclear. According to crystallographic and biochemical studies, one mechanism of Src PTK regulation operates through changes in phosphorylation of their activatory and C-terminal inhibitory tyrosine residues [13], [14]. Lck regulatory tyrosines are C-terminal Tyr505 and Tyr394 situated in the activation loop of the kinase domain. Phosphorylation of Tyr505 by the tyrosine kinase Csk ensures an intramolecular interaction between the C-terminal tail and the SH2 domain of Lck that contributes to an inactive conformation of the enzyme [15]. In contrast, autophosphorylation of Tyr394 correlates with Lck activity [16]. Similar sites regulate Fyn activity. Overall, an equilibrium of conformations ranging between a closed inactive and an open active kinase form is thought to result from the levels of phosphorylation of the regulatory tyrosines [3], [17].

Csk and the tyrosine phosphatase CD45 control in opposite manner the activation state of Lck and Fyn. CD45 is a major positive regulator as shown by the hyperphosphorylation of the inhibitory Tyr and the decreased activities of Lck and Fyn as well as the defective TCR signaling in CD45-deficient T cells [18], [19]. In fact, high expression of CD45 can lead to the dephosphorylation of the Lck activatory Tyr394 [20] and localization of CD45 likely controls Lck activation state [21], [22], [23]. In opposition to CD45, Csk negatively regulates Lck and Fyn, as shown by their 2-5-fold increased basal catalytic activities in Csk-conditional deficient thymocytes [24]. Similarly, human CD4^+^ T cells knocked-down for Csk display a 4-5-fold increased basal phosphorylation of the Lck activatory Tyr394 and a reduced phosphorylation of the inhibitory Tyr505, resulting in faster and stronger TCR-induced activation events [25].

Another mechanism of Src PTK regulation can be allosteric. Thus, the binding of ligands to the SH2 and SH3 domains has been proposed to displace intramolecular interactions and to favor an active conformation that may be further stabilized by phosphorylation of the activatory tyrosine [13], [14]. This model is supported by the enhanced kinase activity of Lck mutants deleted of SH3 and SH2 sequences [15] and of Hck upon displacement of its SH3 domain [26]. Moreover, the SH2 and SH3 domains of Lck and Fyn play an important role in the localization of the kinases by interacting with binding partners. Among these, adaptor proteins appear to regulate Lck and Fyn in a more or less complex manner. An example concerns the regulatory loop involving the transmembrane adaptor protein PAG situated in membrane lipid rafts. In unstimulated T cells, PAG is phosphorylated by Fyn, thereby promoting Csk recruitment and inhibition of Lck. Upon TCR stimulation, Lck has been proposed to be activated as a consequence of PAG dephosphorylation and Csk release [12]. However, the phenotype of PAG-deficient mice indicates the existence of other regulatory or compensatory mechanisms [27]. Indeed, adaptor proteins other than PAG and other proteins can influence Lck activation state [9].

Here, we have studied the relationship between the phosphorylation state of Lck, TCR signal initiation and the transmembrane LAT adaptor in human CD4^+^ T cells. Our data indicate that a substantial pool of Lck and Fyn is basally active in naïve CD4^+^ T cells. Moreover, we provide evidence for the existence of a positive feedback loop between LAT and Lck that promotes TCR signal initiation and that depends on the strength of TCR engagement.

## Results

### Lck and Fyn phosphorylation state in primary T cells

**Figure 1:**
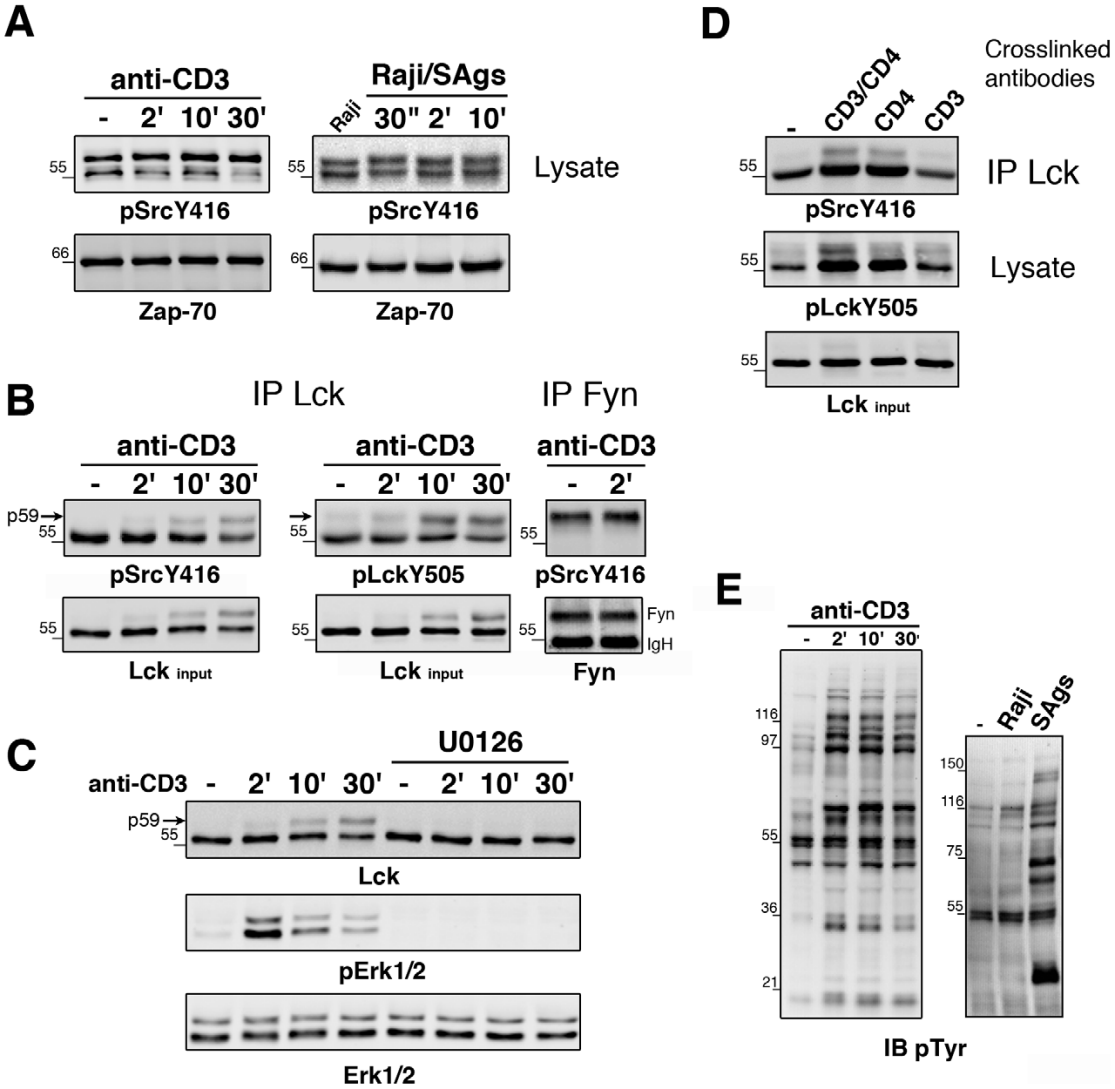
Lck and Fyn phosphorylation state in primary T cells. (A) Naïve CD4^+^CD45RA^+^ T cells were stimulated with anti-CD3, superantigen-pulsed APCs (Raji/SAgs) for the indicated times. Cell lysates were immunoblotted with anti-pSrcY416 and, to control protein loading, with anti-Zap-70. (B) Lck and Fyn immunoprecipitates (IP) from anti-CD3 stimulated cells were immunoblotted with anti-pSrcY416. Phosphorylation of Lck inhibitory Tyr505 was analyzed on separate IP. Lck input corresponds to 10 µg proteins. (C) Immunoblot analysis of Lck IPs, phosphorylated Erk-1/2 and Erk-1/2 in cell lysates of naïve CD4^+^ T cells preincubated or not with U0126 and stimulated with anti-CD3 (one donor used in B). (D) Naïve CD4^+^ T cells were stimulated by crosslinking the indicated antibodies for 30 sec. Lck IPs and cell lysates were immunoblotted with anti-pSrcY416 and anti-pLckY505, respectively. (E) Tyrosine phosphorylations induced by anti-CD3 and Raji/SAgs (2 min) stimulation after immunoblotting cell lysates with anti-phosphotyrosine antibodies. These assays were performed with 9 donors.

We investigated the phosphorylation state of Lck and Fyn in naïve CD4^+^ T cells, making the assumption that upon TCR stimulation these kinases would be phosphorylated on their activatory Tyr and dephosphorylated on their C-terminal inhibitory Tyr. Due to myristoylation and palmitoylation, Lck and Fyn are mainly localized to the inner leaflet of the plasma membrane and in lipid rafts. Therefore, we used dodecyl-β-D-maltoside, a detergent that efficiently solubilizes lipid rafts [23]. This approach was coupled to quantifications of phosphorylation signals. Cell lysates from naïve CD4^+^CD45RA^+^ T cells were immunoblotted with anti-pSrcY416 Abs that are directed against the phosphorylated activatory Tyr motif of Src family members (Figure 1A). Unexpectedly, a doublet of ∼56 and 59 kD was basally phosphorylated and did not increase in intensity in T cells stimulated with anti-CD3 Abs or with superantigen (SAg)-pulsed Raji B cells at least for 10 min following TCR stimulation. Similar results were obtained after T cell lysis under denaturating conditions (Figure S1). To verify that the 56/59 kD doublet contained Lck and Fyn, these kinases were immunoprecipitated (Figure 1B). Surprisingly, both PTKs exhibited a marked basal phosphorylation of their activatory Tyr, detected with anti-pY416 Abs, that did not change after TCR stimulation. However, a slower migrating form of Lck (p59) phosphorylated on Tyr394 appeared at about 10 min, becoming more pronounced at 30 min. No variation of Fyn tyrosine phosphorylation was observed at later times of TCR stimulation (data not shown).

Lck was basally phosphorylated also on the inhibitory Tyr505, supporting the view that it was, at least in part, catalytically inactive in unstimulated cells (Figure 1B). However, no early TCR-induced Tyr505 dephosphorylation was detected at 2 min and both p56 and p59 forms of Lck were still phosphorylated on Tyr505 at 10 and 30 min. Thus, a notable change in Lck following anti-CD3 stimulation was the delayed appearance of p59, which was found to represent a sizeable fraction of Lck in Lck immunoblots (∼35–40% at 30 min). Lck p59 and phosphorylation of Erk1/2 were prevented by T cell pretreatment with U0126, an inhibitor of MEK1/2 kinases (Figure 1C). This implicates the MEK1/2-Erk1/2 pathway in the alteration of Lck mobility, which is likeky due to Ser59 phosphorylation by Erk-1/2 [28], [29].

We then analyzed the phosphorylation state of Lck regulatory tyrosines in naïve CD4^+^ T cells strongly stimulated by crosslinking anti-CD3 and/or anti-CD4 with secondary antibodies (Figure 1D). A clear enhancement of phosphorylation of Lck activatory Tyr was induced by co-crosslinking anti-CD4 and anti-CD3 and by crosslinking anti-CD4 alone but not anti-CD3 antibodies alone. Interestingly, a marked increase in the phosphorylation of Lck inhibitory Tyr505 was detected in the same conditions. Such modifications were also noticed upon T cell stimulation with soluble antibodies although to a much lower extent (data not shown). These data underline the upregulating effect of strong CD4 stimulation on the Lck phosphorylation state and confirm initial findings on Lck [30], [31].

Altogether, these data are consistent with the absence of Tyr505 dephosphorylation in TCR-stimulated Jurkat cells [32] and the poor ability of anti-CD3 [31], [33] or antigen-stimulated TCR [17], [31] to activate Lck. Importantly, despite the presence of a basal pool of Lck and Fyn phosphorylated on their activatory tyrosine, the global protein tyrosine phosphorylation pattern was not elevated in unstimulated T cells compared to its marked induction upon TCR triggering (Figure 1E). These data further suggest that basally active Lck and Fyn are present in resting T cells and are uncoupled from ITAMs.

### Lck activation and its relationship to the LAT signaling module

**Figure 2:**
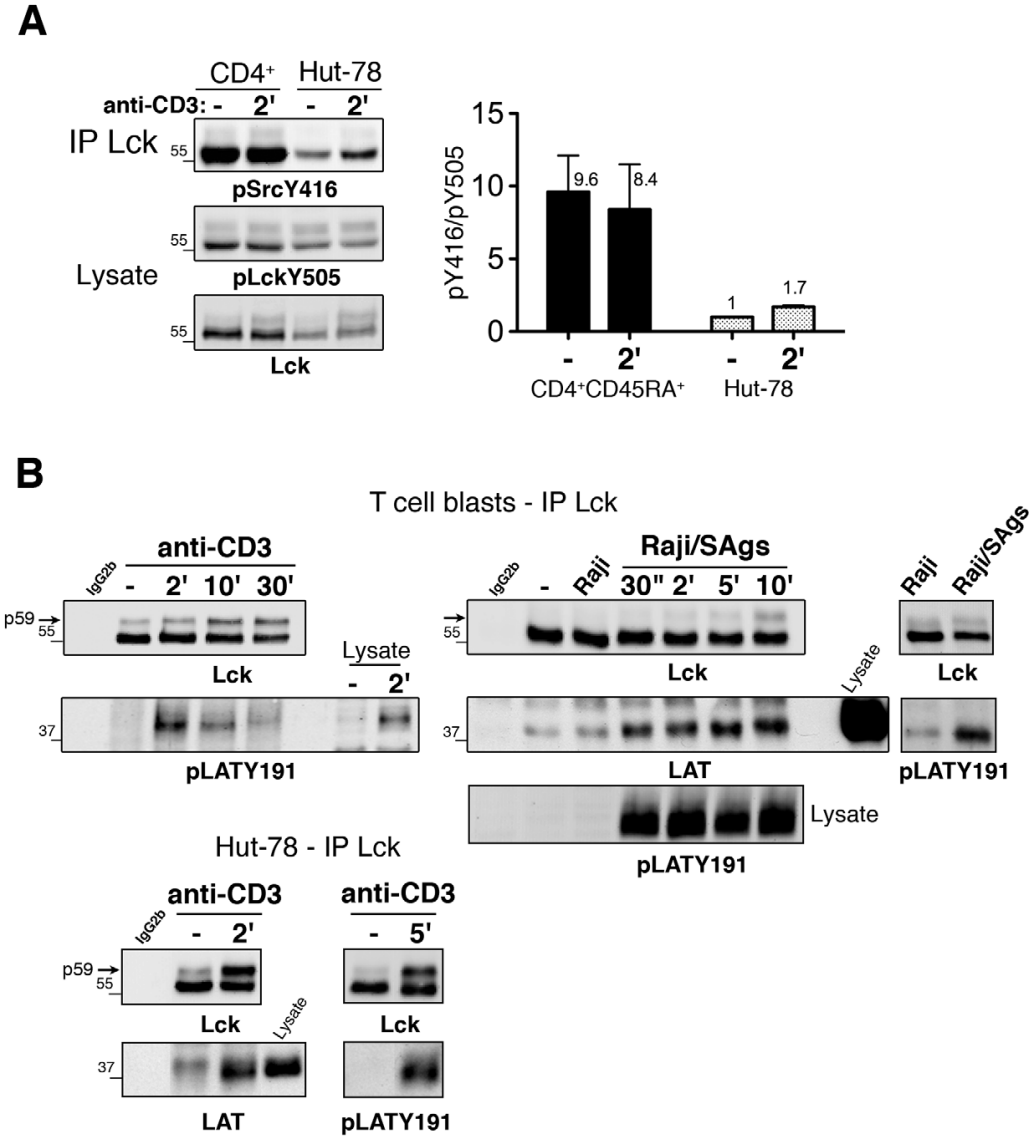
Lck phosphorylation and association to LAT in naïve CD4^+^ T cells and Hut-78 cells. (A) Naïve CD4^+^CD45RA^+^ T cells and Hut-78 cells were stimulated with anti-CD3 for 2 min. Left panel, Lck IPs (150 µg proteins) were analyzed for Tyr394 phosphorylation by immunoblotting with anti-pSrcY416. An aliquot of cell lysates was probed with anti-pLckY505 then with anti-Lck. The histogram shows the mean ratio of phospho-LckY394/phospho-LckY505 +/− SD, from three donors, after normalization of signals to Lck. (B) Lck IPs from T cell blasts stimulated for the indicated times with anti-CD3 or Raji/SAgs (2 min, right panels) were analyzed for LAT co-precipitation by immunoblotting with anti-pLAT-Y191 (left and right panels) and anti-LAT (middle panels). The kinetics of LAT phosphorylation on Tyr191 is shown in cell lysates from superantigen-stimulated T cells. Lck IPs from Hut-78 cells stimulated by CD3 crosslinking were probed with anti-LAT and anti- pLAT-Y191 (lower panels). IgG2b corresponds to lysates from TCR-stimulated cells for 2 min incubated with beads coated with isotype control IgG2b. Assays in B were performed with six donors and at least three times with Hut-78 cells.

We next assessed the phosphorylation state of Lck in the Hut-78 T cell line. We and others found that these cells are more comparable to primary T cells than Jurkat cells in TCR-inducible phosphorylations [8], [34]. Moreover, in Hut-78 cells and in contrast to the Jurkat T cell line, the Lck/Fyn expression ratio is similar to that of primary T cells despite lower levels of Lck and Fyn in Hut-78 cells (data not shown and Figure 2A). In unstimulated Hut-78 cells, immunoprecipitated Lck was phosphorylated on the activatory Tyr394 but to a much lower extent than in naïve CD4^+^ T cells (Figure 2A, left panel). Notably, a clear augmentation of phosphorylated Lck Tyr394 was observed in TCR-stimulated Hut-78 cells. As in primary T cells, in Hut-78 cells, Lck Tyr505 was basally phosphorylated and not dephosphorylated upon TCR stimulation. Normalization of data to the level of Lck revealed a ∼1.7-fold increase in the pTyr394/pTyr505 ratio in TCR-stimulated Hut-78 cells (Figure 2A, right panel). Strikingly, the pTyr394/pTyr505 ratio was ∼9-fold higher in resting and TCR-stimulated naïve T cells than in Hut-78 cells.

We then assessed the capacity of Lck to co-immunoprecipitate with the LAT signaling module. Indeed Lck and its open active form were reported to associate with LAT in Jurkat cells [35], [36]. T cell blasts were stimulated with anti-CD3 and subjected to Lck immunoprecipitation followed by Lck and LAT immunoblotting (Figure 2B, left panels). TCR stimulation led to an increase in Lck p59 shifted form, reaching ∼40% Lck at 30 min (Figure 2B and 1C). Parallel immunoblotting with anti-pLAT-Tyr191 Abs revealed co-precipitated LAT. This Lck/LAT association was also observed with anti-LAT Abs in T cells stimulated with SAgs-Raji cells (middle panels) and in Hut-78 cells (lower panels) in addition to a weak constitutive interaction of the two proteins. Overall, these data indicate that naïve CD4^+^ T cells contain a substantial amount of Lck phosphorylated on the activatory Tyr. Moreover, in both primary T and Hut-78 cells, TCR stimulation leads to the delayed phosphorylation of Lck p59, an increased Lck/LAT association and no dephosphorylation of the Lck inhibitory Tyr505.

### LAT promotes phosphorylation of Lck and Fyn activatory tyrosines in Hut-78 cells

**Figure 3:**
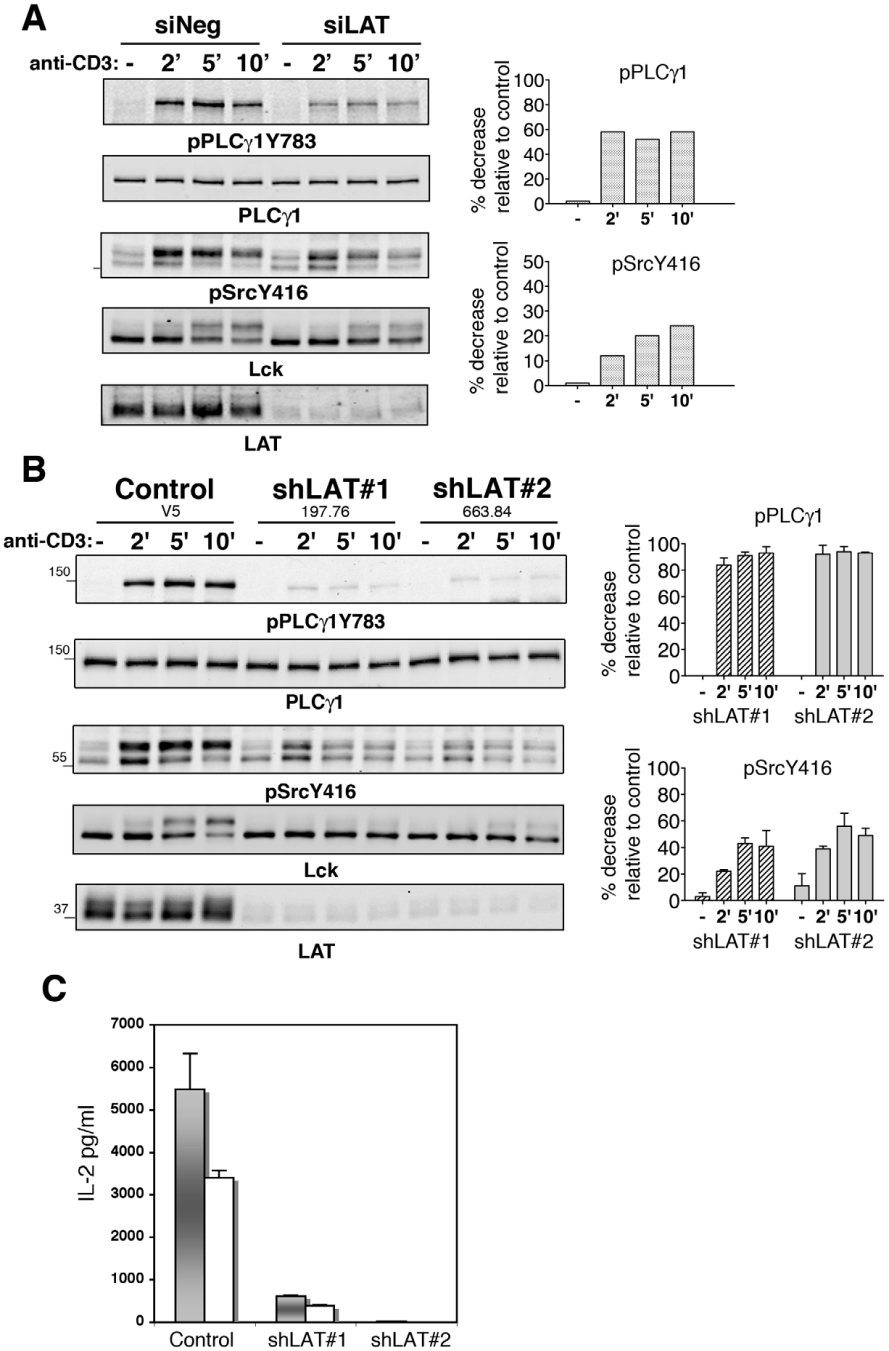
Impaired TCR-induced phosphorylations in Hut-78 cells knocked-down for LAT expression. (A) Hut-78 cells, transiently transfected with siNeg and siLAT RNA, were stimulated with anti-CD3. Cell lysates were immunoblotted with phosphospecific Abs and anti-LAT, then reprobed with anti-PLCγ1 and anti-Lck. Histograms represent inhibition (%) of phosphospecific signals in Hut-shLAT cells relatively to control cells for each time point, calculated after normalization to protein loading. LAT knockdown efficiency was ∼80%. (B) Cell lysates from anti-CD3-stimulated Hut-78 cells transfected with vector (control) and stably knocked-down for LAT expression (∼95%) were immunoblotted as in A. Histograms represent mean percentages +/− SD from three experiments. (C) IL-2 secretion by the indicated cells stimulated with anti-CD3 (0.5 and 0.15 µg/ml, grey and white bars) for 24 h. Data were normalized to PMA/A23187 stimulation. All data are representative of at least three independent experiments.

The TCR-induced co-precipitation of Lck and LAT prompted us to examine the potential role of LAT in TCR signal initiation. To this end, we analyzed the phosphorylation state of Lck and Fyn upon transient RNAi of LAT expression (Figure 3A). Hut-78 cells, transfected with siRNA against LAT (siLAT), exhibited ∼80% LAT knockdown efficiency and ∼ a 60% reduction in TCR-mediated phosphorylation of PLCγ1, a direct partner of LAT [6], [7]. Cell lysates were immunoblotted with anti-pSrcY416 Abs. In TCR-stimulated control cells (siNeg), the level of basally phosphorylated Lck/Fyn doublet increased at 2 min, with the phosphorylated p59 form persisting at 5 and 10 min. Surprisingly, in TCR-stimulated LAT knocked-down cells, the phosphorylation of Lck/Fyn doublet was decreased of ∼10-25% (Figure 3A, right histogram). To achieve higher LAT silencing, Hut-78 cells were stably knocked-down. Two Hut-shLAT clones (shLAT#1, shLAT#2) were severely depleted of LAT (95%) and expressed similar levels of CD3, CD4 and CD45 to control cells (Figure 3B and data not shown). TCR-induced IL-2 secretion (Figure 3C) and PLCγ1 phosphorylation (Figure 3B) were markedly affected in Hut-shLAT cells compared to control cells. Notably, at each time point, the inducible phosphorylation of Lck/Fyn doublet, detected with anti-pSrcY416 Abs, was decreased. This reduction ranged from 20 to 40% in shLAT#1 and 40 to 60% in shLAT#2 cells and was more pronounced at 5–10 min (Figure 3B, right histogram).

**Figure 4:**
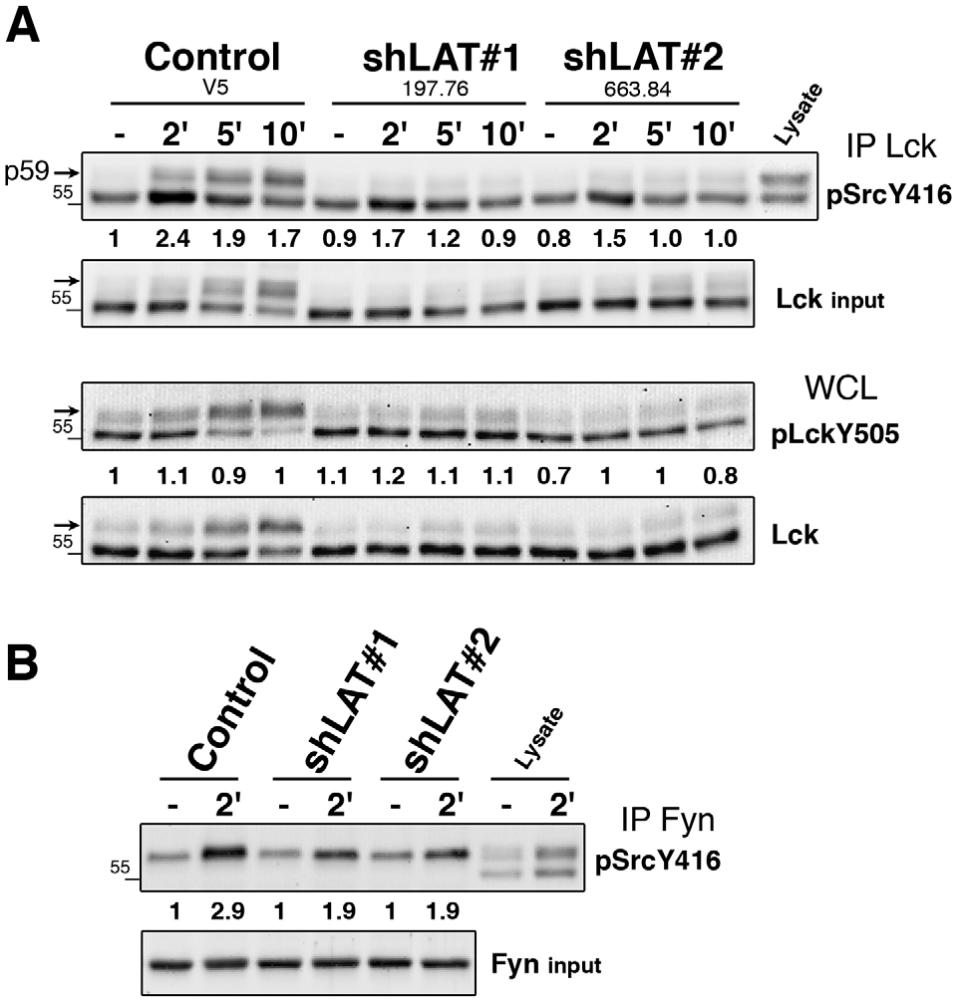
LAT promotes phosphorylation of Lck and Fyn activatory tyrosines. (A) Lck immunoprecipitates from control and Hut-shLAT cells stimulated with anti-CD3 were analyzed for Tyr394 phosphorylation by immunoblotting with anti-pSrcY416. Aliquots of cell lysates were probed with anti-Lck (Lck input) and with anti-LckY505. (B) Fyn immunoprecipitates were analyzed for Tyr417 phosphorylation with anti-pSrcY416. Numbers indicate fold inductions of phosphorylation after normalization to protein loading and unstimulated control cells. Data are representative of three independent experiments.

To investigate the effect of LAT depletion on Lck and on Fyn, the two kinases were immunoprecipitated (Figure 4). TCR-stimulated control cells exhibited an increase in the phosphorylation of the Lck activatory Tyr394 (p56 and p59) at 2 min, which then declined at 5 and 10 min (Figure 4A). In TCR-stimulated Hut-shLAT cells, Lck showed a reduction of phosphorylated Tyr394. A second effect was the nearly complete disappearance of Lck p59, which was consistent with the markedly inhibited Erk activation in Hut-shLAT cells (Figure S2 and see below). This result was also found in TCR-stimulated LAT-deficient Jurkat cells despite the lack of clear differences in Lck phosphorylation on the activatory tyrosine (Figure S3). Similar to control cells, the overall phosphorylation of Lck Tyr505 did not change in Hut-shLAT cells upon TCR stimulation. A third consequence of LAT depletion was the reduction of Fyn phosphorylation upon TCR stimulation (Figure 4B). Together, these results show that LAT promotes TCR-induced phosphorylation of Lck and Fyn activatory tyrosines and is essential for the Erk-dependent Ser phosphorylation of Lck.

### LAT promotes Lck/Fyn regulation though its tyrosine motifs

**Figure 5:**
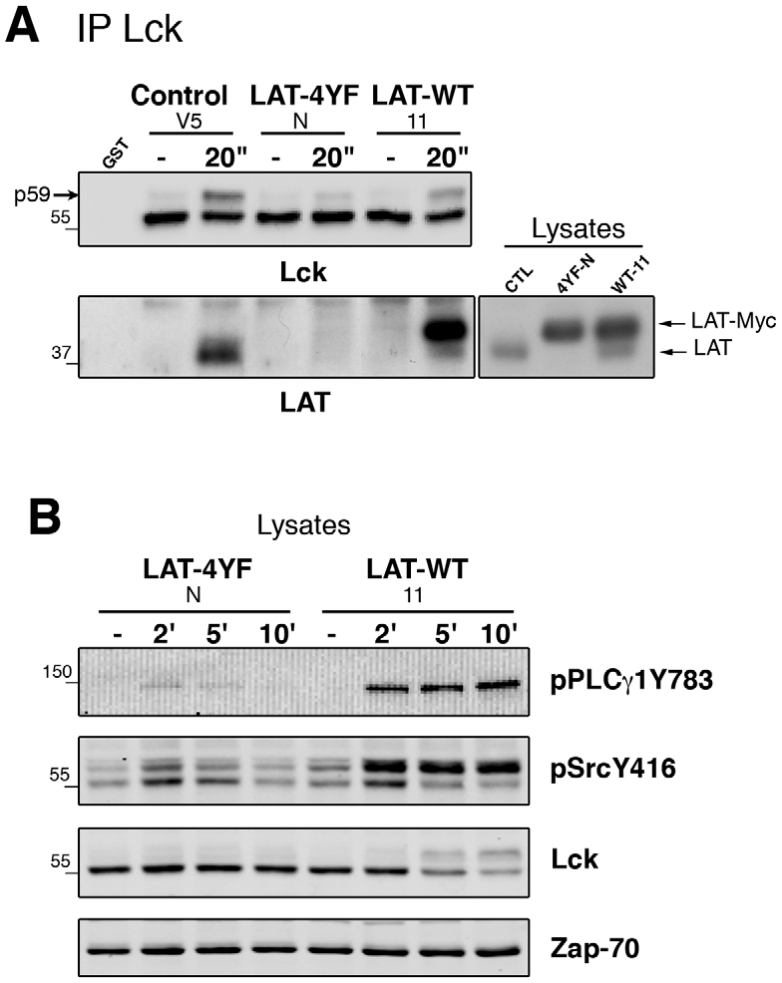
TCR-inducible Lck/LAT association and Lck/Fyn activatory tyrosine phosphorylation depend on LAT tyrosines. (A) Hut-78 cells overexpressing LAT (LAT-WT), LAT-4YF and control cells were stimulated by crosslinking anti-CD3 for 20 s (″). Lck immunoprecipitates were probed with anti-Lck (upper panel) and anti-LAT Abs (lower panel). An aliquot of cell lysates was kept to monitor the expression of endogenous LAT and myc-tagged LAT (right panel). (B) Cell lysates from CD3-stimulated cells were immunoblotted with Abs against pPLCγ1, pSrcY416 and Lck and Zap-70 for protein loading.

To gain insights into the mechanism by which LAT influences TCR signal initiation, we established Hut-78 transfectants overexpressing Myc-tagged LAT-4YF (Hut-LAT-4YF) or LAT (Hut-LAT-WT). The endogenous LAT was silenced in Hut-LAT-4YF cells with lentiviral particles encoding an shRNA targeting LAT but not LAT-4YF. TCR-inducible Lck/LAT association was analyzed in these transfectants. As shown in Figure 5A, upon TCR stimulation, Lck co-immunoprecipitated with LAT in control cells and with endogenous LAT and LAT-myc in Hut-LAT-WT cells. By contrast, the interaction between Lck and LAT-4YF was not observed in Hut-LAT-4YF cells. As previously shown for the LAT-4YF mutant [7], TCR-induced PLCγ1 phosphorylation was strongly decreased (Figure 5B). Notably, the phosphorylation of Lck/Fyn doublet, detected with anti-pY416 Abs, was also reduced in Hut-LAT-4YF compared to Hut-LAT-WT cells. Thus, these data indicate the importance of protein interactions in the mechanism by which LAT controls TCR signal initiation.

### LAT controls TCR signal initiation at low-grade stimulation

**Figure 6:**
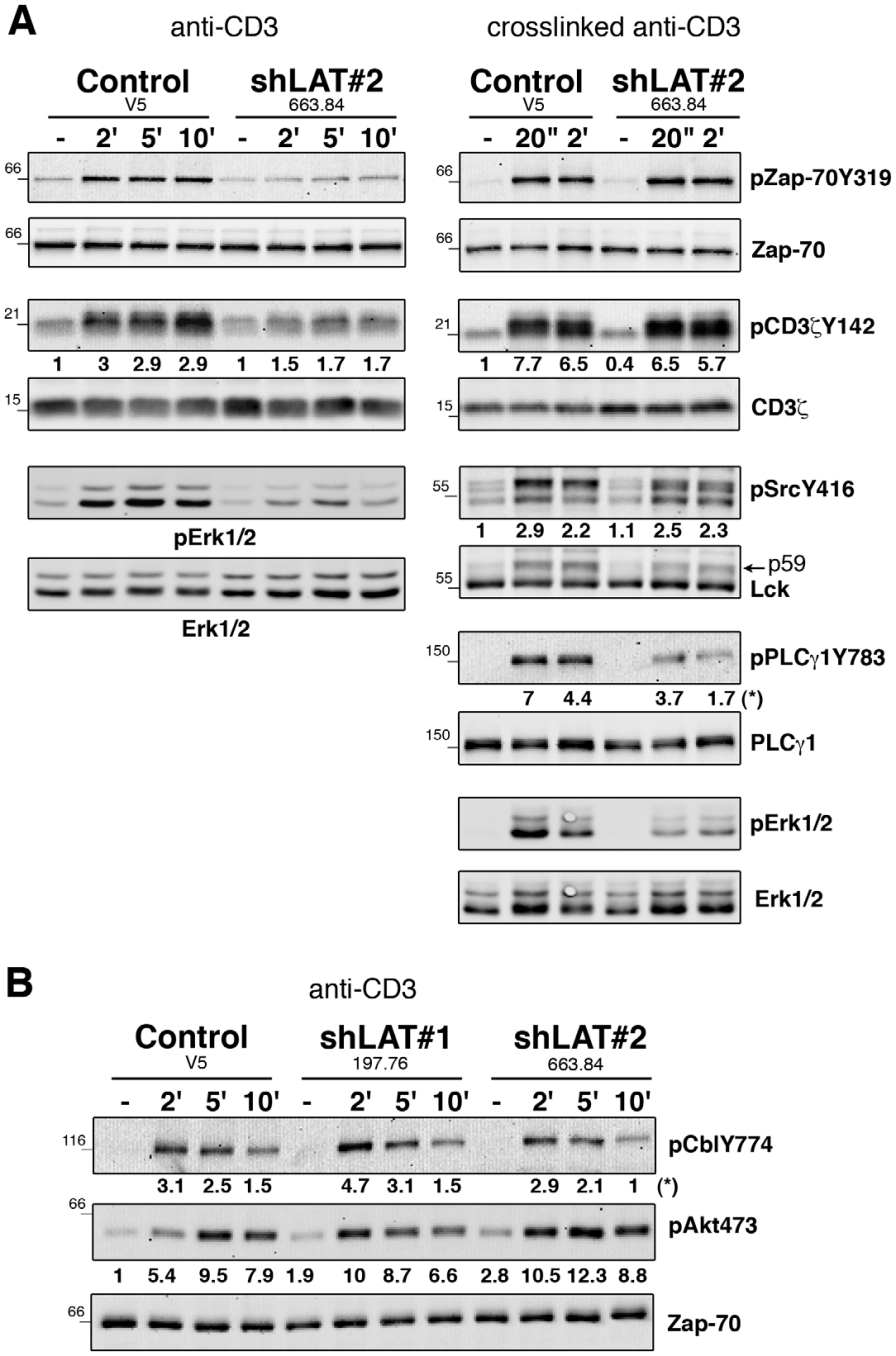
Decreased phosphorylation of Lck substrates in Hut-shLAT cells depends on TCR stimulation strength. (A) Control and Hut-shLAT#2 cells were stimulated with anti-CD3 or by crosslinking anti-CD3 (left and right panels, UCHT1, 5 µg/ml). Cell lysates were analyzed by immunoblotting for phosphorylation of Zap-70, TCRζ chain, SrcY416, PLCγ1 and Erk1/2. Numbers indicate fold inductions of phosphorylation after normalization to protein loading and unstimulated control cells. (*) raw values of phosphorylation after normalization to PLCγ1. Data are representative of at least three independent experiments. (B) Cell lysates from cells stimulated as in A were analyzed for the phosphorylation of c-Cbl and Akt. Data were normalized relatively to Zap-70.

To study the consequences of the altered phosphorylation state of Lck and Fyn in LAT-depleted cells, we analyzed the phosphorylation of direct substrates [4], [5]. TCR-induced Zap-70 and TCRζ chain phosphorylations were markedly impaired in Hut-shLAT#2 cells (Figure 6A, left panels). We also noticed a decrease in TCR-induced Zap-70 phosphorylation in LAT-deficient Jurkat cells in comparison to LAT-reconstituted cells, although this reduction was weaker than that observed in Hut-shLAT cells (Figure S3). Interestingly, the strong TCR stimulation of Hut-shLAT cells induced by CD3 crosslinking resulted in the nearly complete recovery of TCRζ and Zap-70 phosphorylations (Figure 6A, right panels). The Lck/Fyn doublet, detected with anti-pSrcY416 Abs, was also well induced. On the other hand, as shown by Lck immunoblot, the appearance of Lck p59 was still altered most likely as a consequence of the impaired Erk activation. Moreover, PLCγ1 phosphorylation remained strongly decreased in Hut-shLAT cells stimulated by CD3 crosslinking (Figure 6A, right panels and Figure S2).

Importantly, as opposed to the marked defects of TCR signal initiation induced by low-grade stimulation of LAT-depleted Hut-78 cells, the phosphorylation of the ubiquitin protein ligase c-Cbl and of the PI3K-dependent serine/threonine kinase Akt were not decreased in these cells. However, the kinetics of phosphorylation of these two proteins slightly differed in Hut-shLAT and control cells (Figure 6B). Overall, these results show that LAT contributes to the optimal activation of Lck and Fyn and differentially regulates TCR signaling arms.

## Discussion

How the engaged TCR couples to Src kinase-mediated ITAM phosphorylation is an important question that has led to various models mainly based on clustering or conformational changes of the TCR/CD3 complex [37], [38]. In agreement with very recent data [17], [39], we found that unstimulated T cells are equipped with a pool of catalytically active Lck and Fyn that appears to be substantial and not upregulated in response to TCR stimulation in naïve CD4^+^ T cells. For the first time, we provide evidence for a positive feedback loop between LAT and the TCR/Src kinase signaling modules in which LAT promotes TCR signal initiation through active Lck and Fyn. Our data also suggest that LAT may contribute to maintain the active form of Lck close to substrates. In so doing, LAT may sustain not only the initiation but also the propagation of TCR signal.

The finding that, in naïve CD4^+^ T cells, Lck and Fyn were phosphorylated on their activatory Tyr is consistent with the partial phosphorylation of TCRζ chain in resting T cells [40], [41] and the existence of a primed and active forms of Lck, whose level is likely influenced by CD45 and Csk [3], [17], [39]. Transphosphorylation of Lck/Fyn activatory tyrosines may also result from membrane local concentrations (e.g. in lipid rafts) or kinase trapping by pre-clustered TCRs [42]. Our data indicate that the amount of basally active Lck in naïve CD4^+^ T cells is substantial since the Lck pTyr394/pTyr505 ratio was around 9-fold higher than in Hut-78 cells. Despite this marked difference, no massive phosphorylation of TCRζ chain nor of TCR signaling elements occurred in naïve CD4^+^ T cells. An attractive explanation of this may be the sequestration of CD3 ITAMs by phospholipids inside the plasma membrane, rendering them inaccessible to active Lck and Fyn [43]. On the other hand, as recently proposed for Lck [17], the presence of basally active Lck and Fyn, together with the lack of TCR-increased phosphorylation of their activatory tyrosine, suggest that this pool is recruited and utilized by triggered TCRs. Although we cannot exclude the possibility that a minor fraction of kinases may be upregulated upon TCR stimulation in naïve CD4^+^ T cells, it is noteworthy that the TCR-induced Lck p59 form represented a large proportion of Lck (up to 40%). Other studies have reported that TCR stimulation by antigen or with anti-CD3 antibodies is inefficient in enhancing Lck catalytic activity [31], [33], [35], [44], [45] in contrast to the crosslinking TCR and CD4 or CD4 alone [31], [33], [45].

How the pool of active Lck and Fyn may be maintained or upregulated in the vicinity of triggered TCRs is an open question. Interestingly, TCR stimulation of Hut-78 cells led to the enhanced phosphorylation of Lck and Fyn activatory tyrosines. We propose that this may be related to the lower expression and/or the weaker background of phosphorylation of Lck and Fyn in Hut-78 cells compared to primary T cells. Conversely, Jurkat cells express more Lck than Hut-78 cells and exhibit a high basal phosphorylation of Lck activatory Tyr with a very modest enhancement upon TCR stimulation (Figure S3). Our key result, showing that LAT knockdown in Hut-78 cells has an impact on Lck and Fyn activatory tyrosines, strongly suggests that LAT promotes the catalytic activity and/or directs the localization of these kinases. This is also supported by the impaired TCR-mediated TCRζ chain phosphorylation and Zap-70 activation. At a first glance, these data differ from those obtained in LAT-deficient Jurkat cells [6] and in murine LAT-depleted CD4^+^ T cells [46]. This apparent discrepancy may be explained by differences in signal strength. Indeed, when stimulated by crosslinking of anti-CD3 Abs, Hut-shLAT cells behaved similarly to the studied LAT-deficient T cells [6], [46]. In this case, the large recovery of TCR signal initiation may be driven by Lck associated to CD4 [1], [2], [33]. Finally and as reported [6], [46], the absence of LAT did not impair TCR-induced phosphorylation of c-Cbl and Akt. Nevertheless, the kinetics of these events appeared to be modified in Hut-shLAT cells, indicating a complex regulatory role of LAT on TCR signaling.

Interestingly, two binding partners of LAT, the scaffold SLP-76 and the adaptor Grb2 have been recently shown to promote TCR signal initiation and, notably, Zap-70 activation [47], [48]. Although the underlying mechanism and the relation with LAT remain to be detailed, these findings appear to be consistent with our data obtained in LAT-depleted Hut-78 cells.

One mechanism underlying Src activation is the release of SH3 and SH2-mediated intramolecular interactions by ligand binding [3], [14], [15], [26]. Hence, the weak affinity of Lck SH2 for the phosphorylated Tyr505 motif (0.3 µM) was proposed to facilitate the binding of ligands and the Lck transducing ability, even independently of Tyr505 dephosphorylation [49]. Based on this model and on our results showing the critical role of the LAT tyrosines in the Lck/LAT association, we propose that LAT may indirectly maintain active Lck close to substrates and/or stabilize Lck in an open conformation by engaging its SH2 and SH3 domains. Thus, the multimolecular complexes binding to LAT may interact with Lck, thereby promoting Lck concentration and transphosphorylation. In line with this, LAT-Lck co-clusters were reported to depend on protein interactions [50] and Lck binding to SLP-76 was described to positively regulate T cell function [51]. Such role of LAT is reminiscent of the TSAD family adaptor proteins that can modulate Lck catalytic activity and/or localize active Lck to substrates [52]. Alternatively, LAT may regulate Lck by promoting actin-dependent signaling microclusters [53] or its recruitment from an intracellular pool [54]. While such mechanisms may apply to Fyn, the reduction of Fyn activation that we have observed in LAT-depleted cells may be due to defective Lck catalytic activity or localization [45], [55]. Finally, the possibility that Lck phosphorylation by Erk may protect Lck Tyr394 from dephosphorylation by SHP-1 [29] has been examined using U0126 inhibitor (Figure S4) but will require further investigations.

Despite unfavorable conditions of stimulation, such as weak TCR affinity, few ligands on antigen presenting cells and an elevated activation threshold, the TCR signaling machinery is characterized by its high sensitivity and discriminating capacity. The existence of active Src kinases in unstimulated naïve CD4^+^ T cells and of molecular mechanisms maintaining this pool in proximity of the TCR may represent an efficient means to overcome limiting parameters. Notably, the positive control exerted by LAT on TCR signal initiation is sensitive to the strength of TCR engagement. Thus, this mechanism may be critical for T cell outcomes induced by TCR/MHC-peptide interactions of low affinity.

## Materials and Methods

### Ethics statement

Blood samples from healthy donors were from the Etablissement Français du Sang (EFS, Paris) in accordance with a convention between the Institut Pasteur and the EFS. This study was approved according to the DC (Déclaration collective) 2008-68 by the Comité de Protection des Personnes (CPP, Ile de France 1) and Ministère de l'Education Nationale de la Recherche et de Technologie. All donors provided written informed consent for the collection of samples and subsequent analysis.

### Cells and cultures

Hut-78 T [56] and Raji Burkitt cell lymphoma (a gift of Dr. Oreste Acuto, Oxford, UK) were cultured in complete RPMI 1640 containing 10% heat inactivated FCS, 2 mM L-glutamine, penicillin and streptomycin (RPMIc). RPMIc containing 2 µg/ml puromycin was used for Hut-shLAT and control cells, 2 µg/ml puromycin and 2 mg/ml neomycin for Hut-LAT-4YF, 2 mg/ml neomycin for Hut-LAT-WT cells. Primary T cells were maintained in RPMIc with 1 mM sodium pyruvate and non-essential amino acids (GIBCO). Naïve CD4^+^ T cells were purified by negative selection (90–98%) (Miltenyi Biotec). T cell blasts were obtained from phytohemaglutinin (2 µg/ml)-stimulated PBMC for 3 days and then expanded with IL-2 (50 ng/ml, Chiron) during 10–14 days. Resting T cell blasts were starved of IL-2 at least for 48h.

### Antibodies

Mouse antibodies were against CD3 (UCHT1, BioLegend; 1XE, CLB-T3/4.E, Sanquin), CD4 (RPA-T4, eBioscience), Lck (Lck 3A5, Santa Cruz), Fyn (Fyn-01 ascites for immunoblot, a gift from Dr V. Horejsí; Fyn 15 for immunoprecipitation, Santa Cruz), Zap-70 (clone 29, BD Transduction Laboratories), PLCγ1 (clone B-6-4, Upstate), phospho-Thr202/Tyr204-p44/42 MAPK Erk1/2 (clone E10, Cell Signaling), phospho-Tyr142 TCRζ chain (clone K25-407.69, BD Pharmingen), LAT (clone 2E9, Upstate), phosphotyrosine (PY99, Santa Cruz; PY20, Transduction Lab.; 4G10, Upstate) and purified mouse IgG2b used as an isotype control for Lck antibody (a kind gift of P. Bruhns, Institut Pasteur, and from B. Heyman, Uppsala Universitet, Sweden). Rabbit antibodies were against phospho-Tyr416-Src, Tyr505-Lck, Tyr783-PLCγ1, Tyr319-Zap-70, Tyr774-c-Cbl, Ser473-Akt (Cell Signaling), Tyr191-LAT (Biosource) and against Erk1 (clone K23, Santa Cruz). Goat anti-mouse and anti-rabbit IgG coupled to horseradish peroxidase were from Jackson ImmunoResearch laboratories. Goat anti-mouse and goat anti-rabbit IgG coupled to Alexa fluor 680 and DyLight 800 were from Molecular Probes and Pierce, respectively.

### RNA interference and plasmids

Transient knockdown of LAT in Hut-78 cells was achieved using was achieved using siRNA against LAT: 5′-UCUCCAUGGAGUCCAUUGA-3′ and for the negative control siRNA (siNeg): 5′-UAGCGACUAAACACAUCAA-3′ (Dharmacon). Stable LAT knockdown was performed with the sequence-verified short hairpin RNA lentiviral plasmid, pLKO.1-puro, harboring either of the two following sequences, NM_014387-197 5′-CCGGCCTCAGATAGTTTGTATCCAACTCGAGTTGGATACAAACTATCTGAGGTTTTT-3′ and NM_014387-663 5′-CCGGGTCCATTGATGATTACGTGAACTCGAGTTCACGTA ATCATCAATGGACTTTTT-3′. Negative control was the empty pLKO.1-puro vector. pLKO.1 vectors were from Sigma-Aldrich and pCEFL plasmids encoding human LAT Myc tagged were a kind gift from Dr W. Zhang (Durnham, USA) [7].

### Hut-78 cell transfections and transduction

Hut-78 cells were transiently electroporated (12×10^6^/0.4 ml) two times at 24 h intervals with siLAT or siNeg (300 nM), 260 V, 950 µF (Bio-Rad) and used 48 h after the second transfection. Stable Hut-shLAT transfectants were established after electroporation of Hut-78 cells at 230 V, 950 µF with 20 µg pLKO.1-puro shLAT or empty vector (control). Hut-78 cells stably overexpressing LAT-4YF or LAT-WT myc-tagged were obtained by transfection of 20 µg pCEFL vector [7]. Endogenous LAT was silenced in Hut-LAT-4YF cells transduced with lentiviral particles encoding an shRNA against LAT (TRCN0000029735, NM_014387.2-663s, Sigma-Aldrich).

### T cell stimulation, immunoprecipitation and immunoblots

Primary T cells (6–10×10^6^) were stimulated in RPMI (1.5×10^8^/ml) at a ratio of 5:1 with Raji B cells (5×10^7^/ml) pulsed for 1 h with superantigens (Staphylococcal enterotoxin A, B, C3 at 0.2 µg/ml; Toxin Technology) at 37°C. Hut-78 (2–4×10^7^/ml) and primary T cells (1. 2×10^8^/ml) were stimulated with anti-CD3 (5 µg/ml) or by crosslinking of CD3 Abs with anti-IgG_1_ (20 µg/ml) as reported [8]. Cells were lysed at 4°C for 10 min in buffer (20 mM Tris-HCl, pH 7.5, 150 mM NaCl, 1 mM MgCl_2_, 1 mM EGTA, 50 mM NaF, 10 mM Na_4_P_2_O_7_, 1 mM Na_3_VO_4_) containing 1% NP-40 and 1% n dodecyl-β-D-maltoside (Sigma-Aldrich) and inhibitors of proteases. Lck immunoprecipitations were performed for 2 h at 4°C with 3A5 antibodies preadsorbed during 1 h at 4°C to beads from ExactaCruz E kit or directly coupled to agarose (Santa Cruz). Fyn was immunoprecipitated for 2 h at 4°C with Fyn15 Abs preadsorbed to protein G-Sepharose (GE Healthcare). Signals were acquired by ECL and quantified with a Kodak Image Station 440 cf or by immunofluorescence with the Odyssey scanner and Odyssey 1.2 software (Li-Cor Biosciences). Phosphoprotein signals were normalized to the protein loading.

### IL-2 production

Hut-78 cells were stimulated in triplicate at 2.5×10^5^/ml with anti-CD3 (1XE, 0.5 and 0.15 µg/ml) and with PMA (5 ng/ml) and A23187 (0.12 µg/ml) for 24 h at 37°C. IL-2 production was measured by ELISA (R&D Systems).

## Supporting Information

Figure S1 **Lack of TCR-increased phospho-SrcY416 signal in primary T cells lysed under denaturating conditions.** T cell blasts were stimulated with anti-CD3 for 2 min and lysed with usual buffer (Cf. materials and methods) or directly denaturated (50 mM Tris-HCl pH 7.4, 0.5% SDS, 1 mM DTT, 5 mM EDTA, 50 mM NaF, 10 mM Na_4_P_2_O_7_, 2 mM Na_3_VO_4_ and inhibitors of proteases) and boiled for 5 min at 95°C. Lysates were clarified by centrifugation at 4°C for 90 min at 26 000 g and analyzed for phosphorylation by immunoblotting with anti-pSrcY416 and anti-pLAT-Y191.
(TIF)

Figure S2 **Recovery of TCR signal initiation in Hut-shLAT#1 cells stimulated by anti-CD3 crosslinking.** Hut-CTL and Hut-shLAT#1 cells were stimulated by crosslinking of anti-CD3. Cell lysates were analyzed for phosphorylation of Zap-70, TCRζ, SrcY416, PLCγ1 and Erk1/2 by immunoblotting.
(TIF)

Figure S3 **Phosphorylation state of Lck, Zap-70 and Erk kinases in LAT-deficient and LAT-reconstituted Jurkat cells**. JCaM2 cells (Finco et al., 1998) were stably reconstituted for LAT expression and stimulated with anti-CD3 antibodies for the indicated times. Lck was immunoprecipitated and analyzed for phosphorylation with anti-pSrcY416 Abs. Cell lysates were analyzed for phosphorylation of Zap-70 and Erk1/2.
(TIF)

Figure S4 **Inhibition of Erk1/2 activation does not affect TCR signal initiation.** CD4^+^ CD45RA^+^ T cells were incubated with U0126 (50 µM) for 1 h at 37°C, then stimulated with anti-CD3 for the indicated times at 37°C in the presence of inhibitor. Lck immunoprecipitates were analyzed for Tyr394 phosphorylation by immunoblotting with anti-pSrcY416. Cell lysates were probed with anti-Lck (Lck input), anti-pZapY319 and anti-phosphotyrosine Abs. Data are representative of three donors. Similar data were obtained in Hut-78 cells (not shown).
(TIF)
