# Supplementary figures and images for: T Cell Receptor Signal Initiation Induced by Low-Grade Stimulation Requires the Cooperation of LAT in Human T Cells
Source: PLoS One. 2010 Nov 30;5(11):e15114. doi: 10.1371/journal.pone.0015114 (PMC2994893; doi:10.1371/journal.pone.0015114)

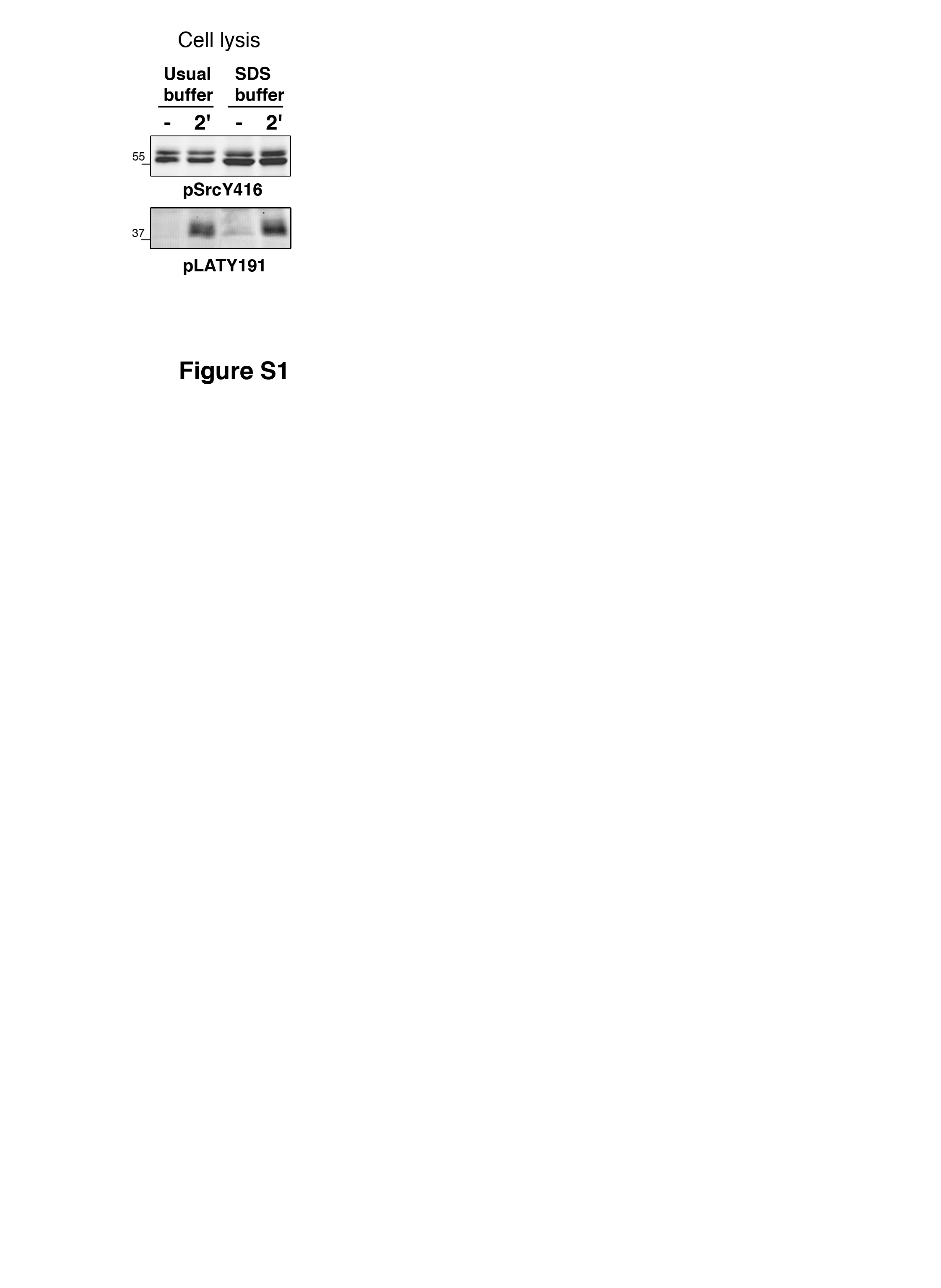

Supplement: Figure S1 — Lack of TCR-increased phospho-SrcY416 signal in primary T cells lysed under denaturating conditions. T cell blasts were stimulated with anti-CD3 for 2 min and lysed with usual buffer (Cf. materials and methods) or directly denaturated (50 mM Tris-HCl pH 7.4, 0.5% SDS, 1 mM DTT, 5 mM EDTA, 50 mM NaF, 10 mM Na4P2O7, 2 mM Na3VO4 and inhibitors of proteases) and boiled for 5 min at 95°C. Lysates were clarified by centrifugation at 4°C for 90 min at 26 000 g and analyzed for phosphorylation by immunoblotting with anti-pSrcY416 and anti-pLAT-Y191. (TIF) [file pone.0015114.s001.tif]

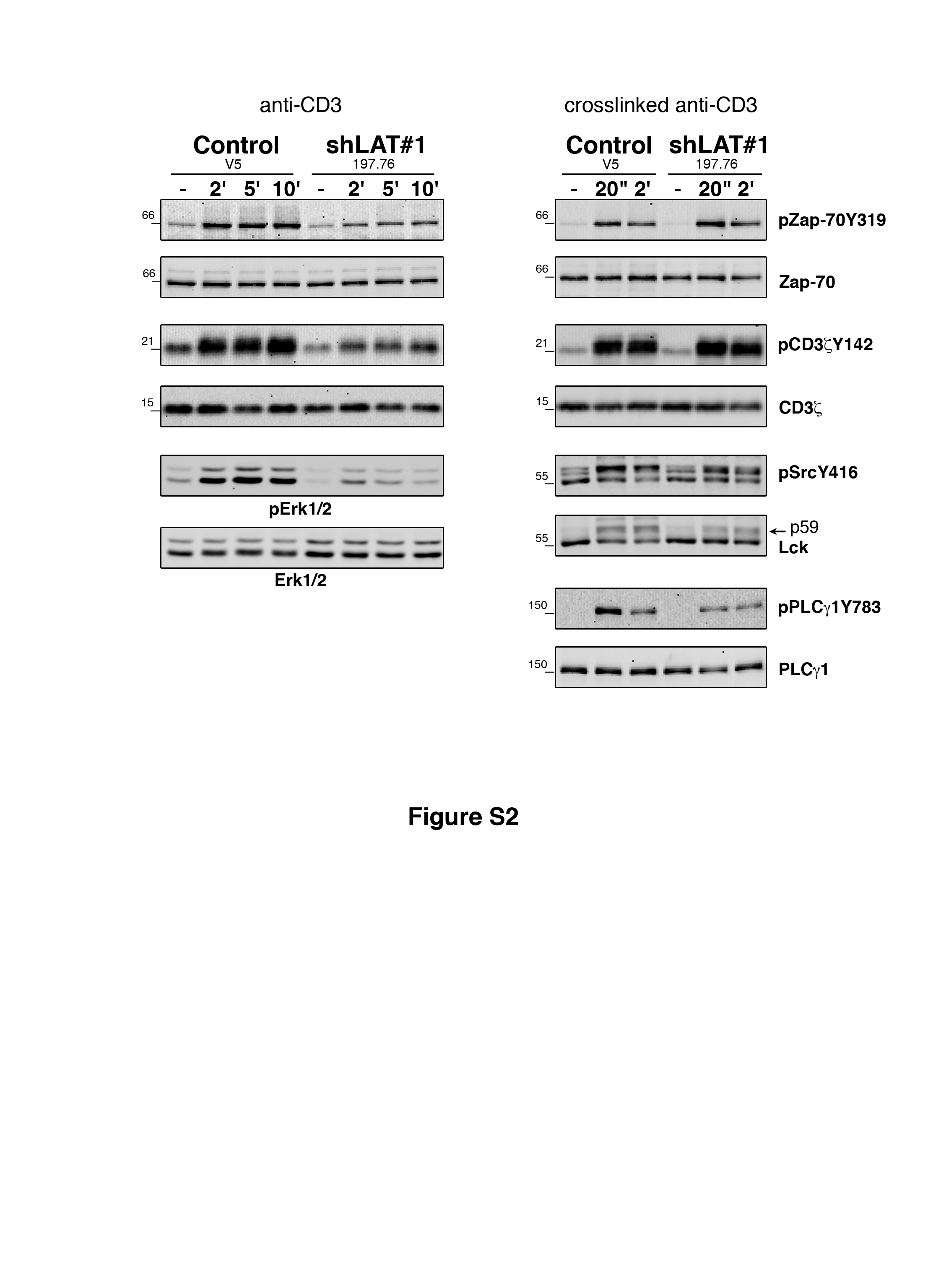

Supplement: Figure S2 — Recovery of TCR signal initiation in Hut-shLAT#1 cells stimulated by anti-CD3 crosslinking. Hut-CTL and Hut-shLAT#1 cells were stimulated by crosslinking of anti-CD3. Cell lysates were analyzed for phosphorylation of Zap-70, TCRζ, SrcY416, PLCγ1 and Erk1/2 by immunoblotting. (TIF) [file pone.0015114.s002.tif]

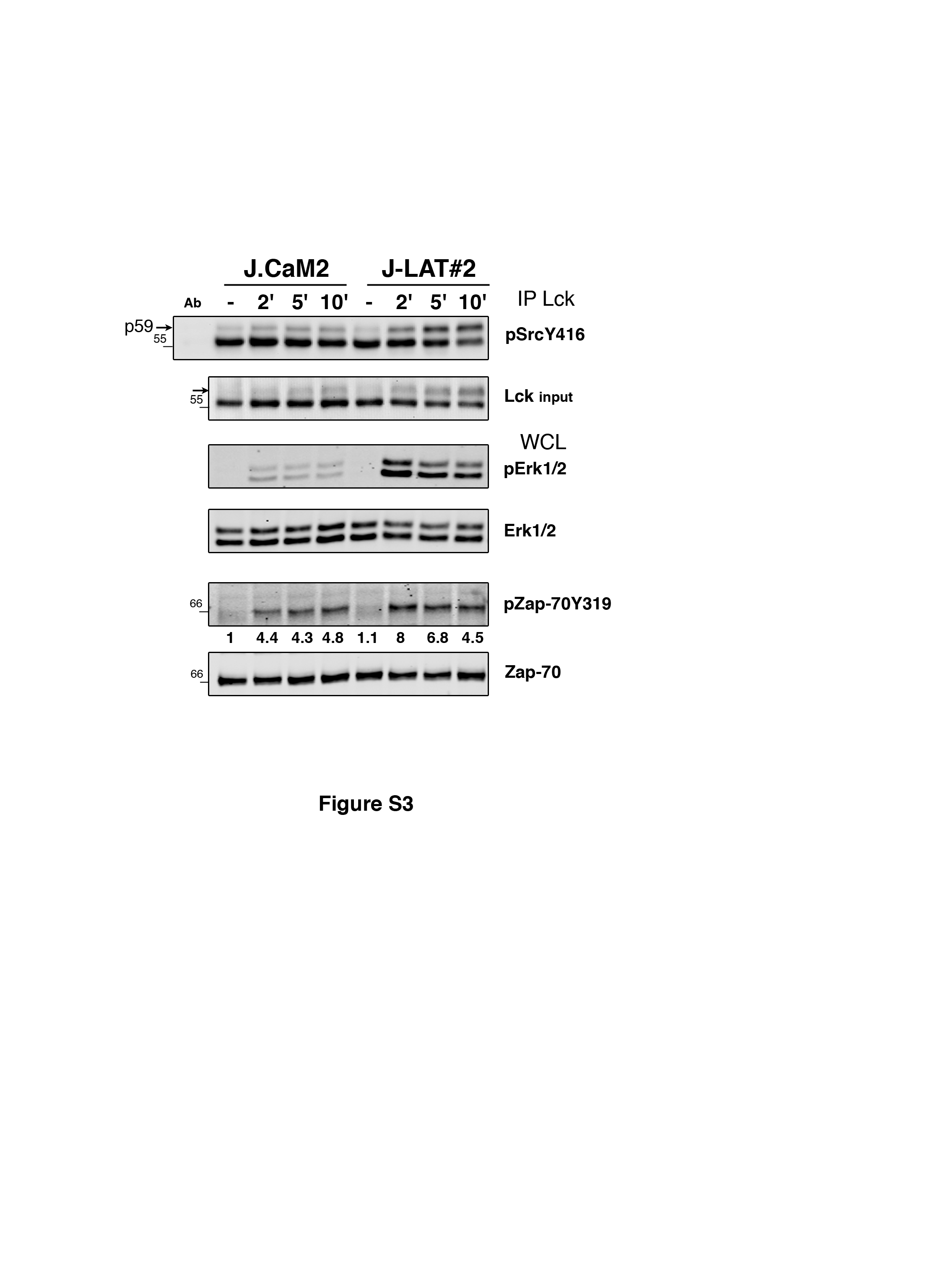

Supplement: Figure S3 — Phosphorylation state of Lck, Zap-70 and Erk kinases in LAT-deficient and LAT-reconstituted Jurkat cells. JCaM2 cells (Finco et al., 1998) were stably reconstituted for LAT expression and stimulated with anti-CD3 antibodies for the indicated times. Lck was immunoprecipitated and analyzed for phosphorylation with anti-pSrcY416 Abs. Cell lysates were analyzed for phosphorylation of Zap-70 and Erk1/2. (TIF) [file pone.0015114.s003.tif]

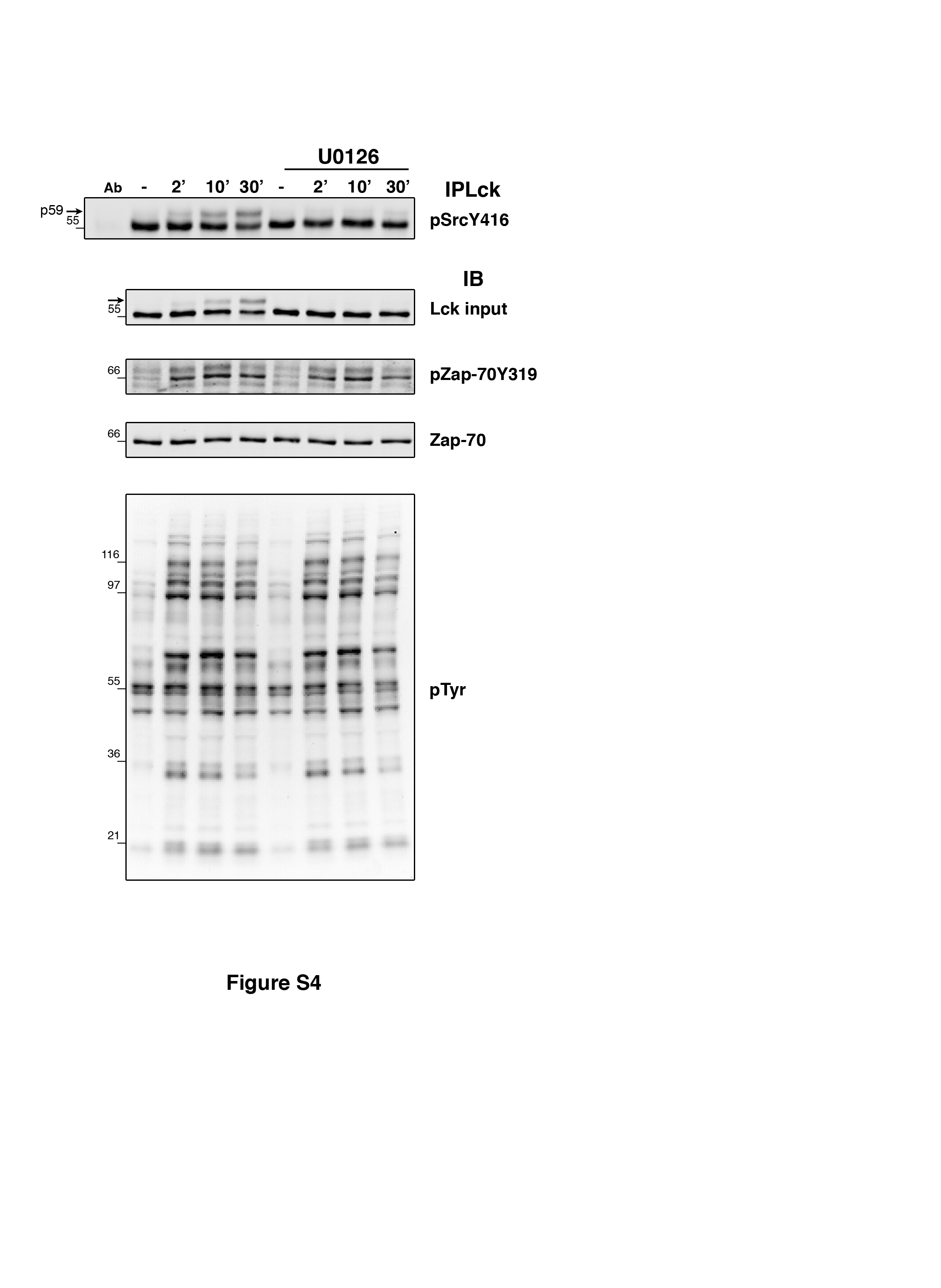

Supplement: Figure S4 — Inhibition of Erk1/2 activation does not affect TCR signal initiation. CD4+ CD45RA+ T cells were incubated with U0126 (50 µM) for 1 h at 37°C, then stimulated with anti-CD3 for the indicated times at 37°C in the presence of inhibitor. Lck immunoprecipitates were analyzed for Tyr394 phosphorylation by immunoblotting with anti-pSrcY416. Cell lysates were probed with anti-Lck (Lck input), anti-pZapY319 and anti-phosphotyrosine Abs. Data are representative of three donors. Similar data were obtained in Hut-78 cells (not shown). (TIF) [file pone.0015114.s004.tif]
